# Previous Education, Sociodemographic Characteristics, and Nursing Cumulative Grade Point Average as Predictors of Success in Nursing Licensure Examinations

**DOI:** 10.1155/2015/682479

**Published:** 2015-10-08

**Authors:** Isaac Amankwaa, Anabella Agyemang-Dankwah, Daniel Boateng

**Affiliations:** ^1^Department of Nursing, Garden City University College, Kumasi, Ghana; ^2^Seventh-day Adventist Hospital, Kumasi, Ghana; ^3^Department of Community Health, Kwame Nkrumah University of Science and Technology, Kumasi, Ghana

## Abstract

*Introduction*. Success in the licensure examination is the only legal prerequisite to practice as a nurse in Ghana. However, a large percentage of nursing students who sit fail this examination for the first time. This study sought to unravel whether prior education, sociodemographic characteristics, and nursing Cumulative Grade Point Average (CGPA) could predict performance in the licensure examinations. *Methods*. The study was a descriptive cross-sectional survey conducted from November 2014 to April 2015 in the Kumasi metropolis, Ghana on 176 past nursing students. Data was collected using questionnaires and analyzed using SPSS version 22. A logistic regression model was fitted to look at the influence of the explanatory variables on the odds of passing the licensure examinations. All statistical significances were tested at *p* value of <0.05. *Results*. Majority, 56.3%, were females and 86.4% were between the ages of 25 and 31 years. Most of the students (88.6%) entered the nursing training colleges with a WASSCE qualification and 38% read general science. 73.9% passed the licensure examinations and the mean CGPA of the students was 2.89 (SD = 0.37). Sociodemographic characteristics and previous education had no influence on performance in the licensure examinations. CGPA had strong positive relationship with performance in licensure examinations (AOR = 15.27; 95% CI = 6.28, 27.11). *Conclusion*. Students CGPA could be a good predictor of their performance in the licensure examinations. On the other hand, students' sociodemographic and previous educational characteristics might not be important factors to consider in admitting students into the nursing training programme.

## 1. Introduction 

Nursing education in Ghana is regulated by the Nursing and Midwifery Council (NMC) for the purpose of rationalizing the training and education of nurses and midwives and the maintenance and promotion of standards of professional conduct and efficiency. The council was set up under the statutory mandate of the National Redemption Council Decree (NRCD) 117 of the 1972 and L.I. 683 of 1971 [1]. The NMC's mandate as provided in Section 4(2f) of NRCD is “the examination of student nurses and midwives” and the determination of nursing graduates' minimum competence and preparedness to provide safe and effective nursing care.

Student nurses who successfully graduate from nursing programs must pass the licensure examination in order to be registered as nurses.

Currently, the licensure examination is held biannually, in February and August. The examination is in two parts, a practical and a theoretical session. The practical component requires that student nurses utilize the nursing process to identify patients' problems, plan, and intervene. Using an Objective Structured Clinical Examination (OSCE) tool, the student is further observed when intervening in the problems identified. The theoretical component has six papers comprising medical and surgical nursing, mental health nursing, paediatric nursing, public health nursing, and obstetric nursing. Every student pursuing nursing must meet the standards of the NMC by passing each of the six examination papers with a minimum of 50% to be eligible for professional registration by the Nursing Council of Ghana. Success in the licensure examination is therefore the only legal prerequisite to practice as a nurse within Ghana. Failing in any paper means that the candidate must pay the full fees again to retake the examination when next offered.

In recent times, however, a large number of students who sit for the council's licensure examinations (NMC-LE) fail on their first attempt [2]. With a national average of 50% pass rate, governmental efforts in producing quality nurses to address key areas of the millennium development goals appear to be threatened. For example, in 2011, only 38.9% of the 3,223 students who sat for the exams passed and in 2013 only 51.8% of the 2439 candidates passed. The situation is quite pronounced in Ashanti region where only 43.7% of the 437 students presented for the licensure examination passed with one school actually recording 100% failure in the examination [2]. In comparison, in the USA, about 15% of nursing graduates taking the licensure examination for the first time fail while the national average success rate is 84.43% [3]. Because of the value placed on success on NMC-LE for both the student and the programme, it is imperative that nurse educators determine predictors of success in the licensure examination [4].

In 2013, the Nursing and Midwifery Council of Ghana commissioned a team of experts from the universities of education (universities mandated to produce professional educators in Ghana, e.g., University of Education, Winneba) in Ghana to investigate the possible factors that account for the poor performance of the nursing and midwifery trainees in the council's licensing examination. Major findings of the study involved school-related, tutor-related, and student-related factors [1]. Student-related factors identified included lack of effective study and time management skills and poor attitude towards clinical sessions. Key evidence missing in the study was the role of students' demographic variables and graduating GPAs in predicting their performance in the licensure examination. This is a major omission since existing studies [5–8] have demonstrated that these factors can predict academic performance in the licensure examination. Tomul and Polat [9], for example, demonstrated that the type of high school from which students have graduated is a strong predictor of subsequent performance. Sayles et al. [10] in their study on 68 associate degree nursing graduates demonstrated that grade point average (CGPA) of prenursing courses was predictive of success on the NCLEX-RN. Within the Ghanaian context, no study has been reported in literature on predictors of successful performance by nursing graduates on the licensure examination. A generalisation of existing evidence to the Ghanaian setting however remains a grey area to be explored.

Failure to understand these factors prevents the design of remediating interventions to help students who are at risk of failing the licensing examination. Recently, heads of some of the nursing institutions have come into a general consensus to register only students with high CGPAs in the licensure examinations. This decision was however based on their observation that students with good performance in the nursing training school tend to perform better in the licensure examinations. There is currently no empirical evidence on the relationship between nursing institution performance and licensure examination. A study by Stewart et al. [11] to explore the relationship between performance in dental school and performance on a dental licensure examination found a positive association between the two. This study will assess the relationship between nursing training institution performance as well as previous education and sociodemographic characteristics on performance in licensure examinations. The empirical evidence is needed to inform policy to address the current unacceptable failure rate of failure in the licensure examinations. We hypothesized that the nursing training performance, previous education, and background characteristics of nursing students could influence their performance in the licensure examinations. Previous education and sociodemographic factors are referred to together as entry characteristics for the purpose of this study.

## 2. Materials and Methods

### 2.1. Study Design and Setting

This was a descriptive cross-sectional survey conducted from November 2014 to April 2015 in the Kumasi metropolis, Ghana. The study involved students of the Kumasi Nursing and Midwifery Training College and Seventh-Day Adventists (SDA) Nursing and Midwifery Training College. The Kumasi metropolis has a number of health facilities in both the public and private sectors. Notable among them is the Komfo Anokye Teaching Hospital (KATH), which is one of the two (2) national autonomous hospitals, four (4) quasihealth institutions, and five (5) healthcare centres owned by the Church of Christ and the Seventh-Day Adventist Church. In addition, there are over two hundred (200) known private health institutions and thirteen (13) industrial clinics in the metropolis. There are nine (9) Maternal and Child Health (MCH) points and one hundred and nineteen (119) outreach sites.

A total of about fifteen (15) institutions are involved in the training of nurses and midwives in the Ashanti region. The Medical Mission Sisters of the Kumasi Church founded Kumasi Nursing and Midwifery Training College in 1957. Currently, it offers two basic diploma programs, nursing and midwifery. It is affiliated to the Komfo Anokye Teaching Hospital in Ghana and its activities are supervised by the Ministry of Education. The institution is accredited by the National Accreditation Board and now awards direct diploma to students after completing a three-year training programme. The school has a total population of about 1200 students. Seven hundred (700) of these students are pursuing diploma in general nursing course while the rest are studying diploma in midwifery.

The SDA Nurses and Midwifery Training College was established in October 2005 by the Central Ghana Conference of the Seventh-Day Adventist Church with the support from Ministry of Health, Ghana, and Kwadaso S.D.A. Hospital. It is located at Kwadaso, a suburb of Kumasi municipality. The school currently has a total population of 720 students and has produced about 1200 nursing graduates since its inception.

### 2.2. Study Population and Sample

The survey involved all past 178 students from the two institutions who sat for the licensure examinations. This involved 83 students from Kumasi Nursing and Midwifery Training College and 95 students from the SDA Nurses and Midwifery Training College. Two students, one from each facility, could not complete the questionnaire so were excluded from the study.

### 2.3. Data Collection and Analysis

Data for this study were collected mainly through interviewing. Participants were interviewed at their various locations (home or health facility) after prior appointments. Participants signed a consent form after the purpose of the study was explained to them and they agreed to participate in the study. Data collection was done with structured questionnaire, which was pretested to check for clarity, consistency, and acceptability of the questions to respondents. All questionnaires and interview results from the field were checked for completeness and internal errors during data collection.

Data were analyzed using SPSS version 22. The main outcomes of the study were students' performance in the licensure examinations and CGDP at the end of the three-year training. Outcome of Licensure examination was coded as “0 = fail” and “1 = pass.” CGPA was treated as a continuous variable and also categorized into class designations (first class, 3.6–4.0; second class upper, 3.0–3.59; second class lower, 2.5–2.99; third class, 2.0–2.49; pass, 1.5–1.99; and fail, 0–1.49). A test for normality showed an approximately normal distribution of CGPA and therefore was not transformed for the analysis performed. The variables were described using frequencies and percentages. Univariable associations between the independent variables in the study and licensure were tested with chi-square/Fischer's exact while associations with CGPA were tested with one-way analysis of variance (ANOVA). A logistic regression model was fitted to further look at the relationship between CGPA, sociodemographic characteristics, and previous education on the odds of passing the licensure examinations. The variables considered in the model (based on preknowledge of their relationship with students performance) were gender, age, home community, entry category into nursing (Senior Secondary School Certificate Examination, West African Senior School Certificate Examination or mature), course followed at high school (general science or other), high school ownership (government owned or other), academic standard of high school, and career choice of nursing programme (first, second, third, or fourth). All statistical significances were tested at *p* value of <0.05.

## 3. Ethical Consideration

The ethical consideration of confidentiality was strictly upheld in this study. This was done through protection of the privacy of the students by not revealing their identities. The necessary permission was also obtained from ethics committees of selected institutions. Informed consent was sought from prospective study participants. The research protocol was further subjected to review and approval by the Committee for Human Research Publication and Ethics (CHRPE) of KNUST/KATH. Information gathered from participants was kept in a safe place to further ensure confidentiality.

## 4. Results

### 4.1. Background Characteristics and Previous Education of Students

Table 1 presents the background characteristics of the students. Majority, 56.3%, were females whereas 43.7% were males. Most of them (86.4%) were between the ages of 25 and 31 years. More than 90% were Christians and about 36.4% described their home community as city or urban. Majority, 52.2%, described their town as semiurban. About 46% stated that their mothers had basic education and 23.9% stated that their mothers had secondary education whereas 14.2% stated that their mothers had no formal education. With respect to their fathers' educational background, 43.2% had tertiary education whereas 29.5% had secondary education. Only 4% had no formal education.

Most of the students (88.6%) entered the nursing training with a West African Senior School Certificate Examination (WASSCE) qualification whereas only 2.3% entered as mature students. About 38% read general science and 47.2% read general arts whereas 12.5% read home economics. Majority of the students entered the nursing training from government owned institutions (73.3%) whereas 16.5% and 10.2% were from mission and private institutions, respectively. Majority of the students described their former schools as in urban setting (68.6%) and had high academic status (68.2%). One hundred and eighteen students constituting 67% chose nursing first whereas it was second choice for 23.3% of the students. Only 3.4% and 2.8% of respondents' mothers and fathers were health workers, respectively.

The students had diverse motivations for choosing the nursing profession. This was assessed with a multiple response question and the most cited reason by the students was “always wanted to be a professional nurse,” 55.1%. Having the desire to help others was the second (49.1%) and third most cited reason for pursuing the nursing profession was opportunity of getting job offer after completion, 21%. Other reasons included seeing the nursing as a secure job (25%), availability of students' allowances (8%) and exposure to family and friends in healthcare profession (7.4%).

### 4.2. Influence of Background Characteristics and Previous Education on Licensure Examinations

As shown in Table 2, one hundred and thirty students representing 73.9% passed the licensure examinations whereas 46 (26.1%) failed. The mean CGPA of the students was 2.89 (SD = 0.37). When grouped into class designations, most of the students had second class division with 31.2% in upper division and 48.9% in the lower division. Twenty-four students (13.6%) had third class whereas no student failed. There was a strong association between CGPA and performance in the licensure examinations with the percentage of students who passed decreasing down the class designations. All the students who had first class passed whereas only 45.8% of those who had third class passed the licensure examinations.

The CGPA of the students was significantly associated with the performance in the licensure examinations with the mean CGPA being higher among those who passed the licensure examination as compared to those who failed (2.98 versus 2.63; *p* < 0.001). A univariable logistic regression analysis showed increased odds of passing the licensure examination with increasing CGPA of the students (OR = 19.47; 95% CI = 7.02, 32.96).

Table 3 shows results of the influence of background characteristics on their CGPA and performance in the licensure examinations. The univariable analyses indicate that the sociodemographic background of the students had no influence on their cumulative performance in the nursing training as well as the licensure examination. The percentage of students who passed the licensure examinations was not significantly different between the various categories for all the demographic variables studied.

As shown in Table 4, the univariable analysis of the students' previous education also showed no influence on students' performance in the licensure examinations. Similar to the influence of sociodemographic characteristics, the differences in percentage of students who passed the licensure examination between the various categories of previous education variables studied could not reach significant levels. With respect to CGPAs of the students, the type of former school attended had significant influence on the CGPA with mean CGPA being highest among those who attended government schools followed by mission schools.

### 4.3. Multivariable Analysis

In the multivariable analysis, CGPA had strong positive relationship with performance in licensure examinations after adjusting for the sociodemographic characteristics and previous education (AOR = 15.27; 95% CI = 6.28, 27.11). Age, gender, home residence, entering nursing training with WASSCE, parental education, nursing being a first choice career of choice, and reading science in formal school were entered in the model. As shown in Table 5, the relationship between these covariates and performance in licensure examination was however not statistically significant. This indicated that the performance of students in the licensure examination could not be explained by the sociodemographic characteristics and previous education factors considered in this study.

## 5. Discussion 

Nursing education should be taken as an important aspect of healthcare provision, by ensuring nurses with the right training and characters are produced to provide the necessary healthcare. Writing and passing the licensure examination is required for registration and practice as a nurse. This study sought to investigate the relationship between school performance and licensure examinations. Finding from this study shows a very strong positive relationship between school CGPA and performance in the licensure examinations. This is supported by studies from Stewart et al. [11] and Ranney et al. [12] which both found a significant association between school performance and performance in the licensure examination. Similarly, existing studies have reported a strong correlation between students' previous academic achievement and their success in NCLEX-RN examination [13–15]. The issue however is debatable on the basis of how right it is to refuse a student who has completed the nursing training from sitting for the licensure examinations. We believe that participation in the licensure examination should be based on progress in the nursing training and not just academic performance.

While NCLEX-RN examination is designed to identify candidates who possess the theoretical knowledge to practice safely as an entry-level nurse, NLE measures nursing students' academic outcomes and practical skills. The current study therefore extends the existing evidence (strong correlation between academic success and prior academic achievement) to include an association between students' previous achievement and their psychomotor skills. Future studies must however explore the content validity of nursing licensing examinations. This will ensure that nurses not only are competent academically but also possess some additional professional qualities.

Nonacademic or demographic characteristics of students have been cited as important variables in predicting academic performance of the students [16–18]. The understanding of the relationship between student-related factors and academic performance and predictors of student's success in nursing schools is important for not only the nursing institutions but also students, parents, and the society as a whole. This will enable parents and counselors to give students better-informed advice on choosing their college programmes, and students are able to better understand their potentials and make wiser decisions for their future. This study sought to investigate the sociodemographic and previous education factors that influence students' performance at the Nursing and Midwifery Training College. The input as used in this model refers to the background characteristics of the student, including family background. Majority of the respondents were females and were between the ages of 25 and 31 years. The outcome is identified as the students' performance in the licensure examinations. Findings from this study suggest that sociodemographic and previous education characteristics of nursing students play no role in their academic performance.

Contrary to previous research, the study did not find any association between age, gender, and academic performance of the students [19, 20]. This study finding is inconsistent with findings from previous researchers who assert that female students perform better than the male students [19, 21, 22]. This suggests that despite the gender or age of the students, they stand an equal chance of performing well in the nursing training programme and the licensure examination.

This study found no influence of parents' education on performance in the licensure examinations. This is inconsistent to other previous studies, which found parental education as well as employment background as important factor that significantly affects students' academic success [23, 24]. Melby and Conger [25] have also opined that family income, parental education, and parental involvement have an influence on the academic experience of students in their postsecondary education. Based on previous researches on relationship between parents' educational background and students' performance [26], the students whose parents had higher educational levels would be expected to perform better. As indicated by McMillan and Western [27], based on the cultural capital theory, students who come from well-educated families will obtain success. This study however found contradictory results in this setting. Although majority of students' parents had high level of education, this had not translated into the level of performance in the licensing examinations and could be as a result of other factors that have greater contribution to performance in the licensing examinations in this setting.

This study again looked at previous academic programme in senior high school and entry category and how these influence performance in licensure examinations. The differences in performance in licensure examinations with respect to previous course at the senior high school could not reach significant levels. Findings from this study suggest that the students course offered at previous educational level and the entry level characteristics may not play an important role in determining the students success in the licensure examination although previous studies have found a significant relationship. Potolsky et al. [28], for instance, found academic performance on the science courses to correlate significantly with nursing school performance in the first year. The study by Tanya [29] also found an influence of preadmission science scores on success in nursing training programme. Our study on the other hand demonstrated that the previous course read by the students does not matter in their performance of the licensure examination.

## 6. Limitations 

This study might however suffer some methodological limitations. The inability to include all or majority of the nursing institutions in the country could influence the generalizability of these study findings. This study is generalizable to the Kumasi metropolis only. It is however assumed that characteristics of students in nursing training institutions do not differ much across the region. The study might have also left out other important characteristics that could influence performance of nursing students in the licensure examinations.

## 7. Conclusion 

The findings of this study suggest that students' previous education and demographic characteristics do not play a role in their performance in the licensure examinations. This further indicates that other school levels as well as tutor factors and issues relating to the programme may better explain the differences in outcome with respect to the licensure examinations. It is recommended that a multilevel study be conducted to explore other factors such as availability of academic resources to enhance learning, students perceptions of the nursing programme, tutors motivation, and students study skills, thinking abilities, and time management. It is further recommended that this study be conducted with the inclusion of other nursing institutions in the region to have a deeper insight into the subject matter. The study however indicates that the school performance of the schools is a good predictor of their performance in the licensure examinations. Efforts to improve students learning and outcome at the school level should therefore be promoted. However, students having a prior notice of school performance as criteria for being selected for licensure examinations could encourage them to put much effort in their study.

## Figures and Tables

**Table 1:** Description of background characteristics and previous education.

Variable	*N* = 176	Percentage
Gender		
Male	77	43.7
Female	99	56.3
Age		
21–25	6	3.4
26–30	152	86.4
31–35	18	10.2
Christian religion	162	92.0
Description of home community		
Rural	20	11.4
Semiurban	92	52.2
City/urban	64	36.4
Mother education		
Non	25	14.2
Basic	81	46.0
Secondary	42	23.9
Tertiary	28	15.9
Father education		
Non	7	4.0
Basic	41	23.2
Secondary	52	29.5
Tertiary	76	43.2
Mother health worker	6	3.4
Father health worker	5	2.8
Entry category into nursing programme		
SSSCE	14	8.0
WASSCE	156	88.6
Mature	6	3.4
Course read during high school education		
General science	67	38.1
General arts	83	47.2
Home economics	22	12.5
Agricultural science	4	2.3
Former school		
Government owned	129	73.3
Mission	29	16.5
Private/other	18	10.2
Former school was located in urban setting	121	68.6
Former school has high academic status	120	68.2
Nursing as a career of choice		
First	118	67.0
Second	41	23.3
Third/fourth	17	9.7

**Table 2:** Relationship between school performance and licensure examination.

School performance	Licensure examination		Total	*p* value
	Passed *n* = 130	Failed *n* = 46		
Mean CGPA, mean (SD)	2.98 ± 0.37	2.63 ± 0.22	2.89 ± 0.37	<0.001
CGPA, *N* (%)				
First class (3.6–4.0)	11 (100.0)	0 (0.0)	11 (6.3)	<0.001
Second class upper (3.0–3.59)	53 (96.4)	2 (3.6)	55 (31.2)	
Second class lower (2.5–2.99)	55 (64.0)	31 (36.0)	86 (48.9)	
Third class (2.0–2.49)	11 (45.8)	13 (54.2)	24 (13.6)	
Total	130 (73.9)	46 (26.1)		

**Table 3:** Influence of sociodemographic characteristics on performance in licensure examinations.

Variable	Licensure exam % passed	*p* value^*∗∗*^	Mean CGPA	*p* value^*∗*^
Gender				
Male	76.6	0.288	2.9 (0.4)	0.451
Female	71.7		2.9 (0.4)	
Age				
21–25	50.0	0.180	2.7 (0.2)	0.361
26–30	76.3		2.9 (0.4)	
31–35	61.1		2.8 (0.4)	
Christian religion	72.8	0.210	2.9 (0.4)	0.143
Description of home community				
Rural	70.0	0.919	2.8 (0.3)	0.062
Semiurban	75.0		2.9 (0.4)	
City/urban	73.0		2.9 (0.4)	
Mother's education				
Non	72.0	0.917	2.9 (0.3)	0.078
Basic	74.1		2.9 (0.4)	
Secondary	71.4		2.9 (0.4)	
Tertiary	78.6		2.9 (0.4)	
Father's education				
Non	71.4	0.796	2.8 (0.3)	0.395
Basic	78.0		2.8 (0.3)	
Secondary	69.2		2.9 (0.3)	
Tertiary	75.0		2.9 (0.4)	

^*∗∗*^Analysis of variance.

^*∗*^Chi-square/Fisher's exact test.

**Table 4:** Influence of previous education on students' performance.

Variables	Licensure exams	*p* value^*∗*^	Mean CGPA	*p* value^*∗∗*^
	% passed			
Entry category into nursing programme				
SSSCE	71.4	0.474	2.82 (0.4)	0.655
WASSCE	73.1		2.90 (0.4)	
Mature	100.0		2.74 (0.3)	
Course read during high school education				
General science	76.1	0.491	2.89 (0.4)	0.828
General arts	73.5		2.89 (0.4)	
Home economics	63.6		2.88 (0.4)	
Agricultural science	75.0		3.06 (0.6)	
Former school was				
Government owned	76.7	0.352	2.93 (0.4)	0.026
Mission	65.5		2.78 (0.4)	
Private/other	66.7		2.74 (0.3)	
Former school was located in urban setting				
Agree	77.7	0.087	2.92 (0.4)	0.460
Disagree	65.5		2.85 (0.4)	
Former school has high academic status				
Agree	75.8	0.364	2.90 (0.4)	0.147
Disagree	69.6		2.91 (0.3)	
Nursing as a career of choice				
First	72.9	0.801	2.87 (0.4)	0.715
Second	78.0		2.93 (0.4)	
Third/fourth	70.6		2.89 (0.4)	

^*∗∗*^Analysis of variance.

^*∗*^Chi-square/Fisher's exact test.

**Table 5:** Logistic regression analysis of influence of entry characteristics on performance in licensing examinations.

Variable	Adjusted odds ratio (95% CI)	SE
CGPA	15.27 [6.28, 27.11]^*∗∗∗*^	0.88
Age <30 years	0.31 [0.06, 1.63]	0.85
Male gender	1.13 [0.50, 2.55]	0.42
Home residence (ref = rural)		
Semiurban	1.49 [0.41, 5.37]	0.65
City/urban	1.44 [0.38, 5.42]	0.68
Father highly educated	0.56 [0.21, 1.49]	0.49
Mother highly education	1.23 [0.55, 2.76]	0.41
Entered nursing with WASSCE	0.32 [0.04, 2.63]	1.08
Nursing was a first career of choice	0.50 [0.21, 1.19]	0.45
Read science in formal school	1.2 [0.5, 2.6]	0.40

^*∗∗∗*^ *p* < 0.0005.
